# Opioid Prescription Patterns and Risk Factors Associated With Opioid Use in the Netherlands

**DOI:** 10.1001/jamanetworkopen.2019.10223

**Published:** 2019-08-28

**Authors:** Ajda Bedene, Willem M. Lijfering, Marieke Niesters, Monique van Velzen, Frits R. Rosendaal, Marcel L. Bouvy, Albert Dahan, Eveline L. A. van Dorp

**Affiliations:** 1 Department of Clinical Epidemiology, Leiden University Medical Center, Leiden, the Netherlands; 2 Department of Anesthesiology, Leiden University Medical Center, Leiden, the Netherlands; 3 Division of Pharmacoepidemiology and Clinical Pharmacology, Utrecht Institute for Pharmaceutical Sciences, University Utrecht, Utrecht, the Netherlands

## Abstract

**Importance:**

An increase in opioid prescription has been observed in the Netherlands. It is vital to understand this increase and to identify risk factors for opioid prescription to ensure that health interventions remain appropriately targeted.

**Objectives:**

To determine the prevalence of opioid prescriptions and adverse events associated with opioids, and to identify risk factors associated with opioid prescription in the Dutch population.

**Design, Setting, and Participants:**

This cohort study used national statistics from the Netherlands from January 1, 2013, to December 31, 2017, including the full Dutch population of 16 779 575 people in 2013 and 17 081 507 people in 2017. Data from the Dutch Health Monitor surveys of 2012 and 2016 were also included. Databases were anonymized prior to analysis. All analyses were performed between December 2018 and February 2019.

**Exposure:**

Opioid prescription.

**Main Outcomes and Measures:**

The main outcomes were the dynamics of opioid prescriptions, hospital admissions for opioid overdose, and opioid overdose mortalities. The secondary outcome was risk factors associated with opioid prescription.

**Results:**

In 2013, 814 211 individuals (4.9% of the total population) received an opioid prescription. In 2017, 1 027 019 individuals (6.0% of the total population) received at least 1 opioid prescription (mean [SD] age, 59.3 [18.5] years; 613 203 [59.7%] women). The rate of hospital admissions for opioid overdose was 9.2 per 100 000 inhabitants in 2013 and 13.1 per 100 000 inhabitants in 2017 (relative risk, 1.43 [95% CI, 1.34-1.52]). Similarly, an increased risk of opioid overdose death was observed, from 0.83 per 100 000 inhabitants in 2013 to 1.2 per 100 000 inhabitants in 2017 (relative risk, 1.49 [95% CI, 1.20-1.85]). Based on data from the 2012 Dutch Health Monitor survey, risk factors associated with opioid prescription included being older than 65 years (odds ratio [OR], 4.20 [95% CI, 3.98-4.43]), having only a primary school education (OR, 3.62 [95% CI, 3.46-3.77]), being widowed (OR, 3.30 [95% CI, 3.13-3.49]), reporting always feeling symptoms of depression (OR, 3.77 [95% CI, 3.41-4.18]), and reporting poor or very poor physical health (OR, 10.40 [95% CI, 10.01-10.81]). Self-reported back pain (OR, 4.34 [95% CI, 4.23-4.46]) and rheumatoid arthritis or fibromyalgia (OR, 3.77 [95% CI, 3.65-3.90]) were also associated with opioid prescription. However, unemployment (OR, 1.05 [95% CI, 0.96-1.13]) was not associated with opioid prescription, and alcohol use disorder (OR, 0.76 [95% CI, 0.73-0.80]) was negatively associated with opioid prescription.

**Conclusions and Relevance:**

This study found that opioid prescriptions have increased in the Netherlands. Although the risk of adverse events is still relatively low, there is an urgent need to review pain management to prevent a further increase in opioid prescription.

## Key Points

**Question:**

Are the prevalence of opioid prescriptions and the incidence of hospital admissions for opioid overdose and opioid overdose mortality changing in the Netherlands through time?

**Findings:**

In this cohort study with nationwide data from the Netherlands, 814 211 individuals (4.9% of the total population) were prescribed an opioid in 2013, and 1 027 019 individuals (6.0% of the total population) were prescribed an opioid in 2017. Hospital admissions for opioid overdose increased from 9.2 per 100 000 inhabitants in 2013 to 13.1 per 100 000 inhabitants in 2017, and opioid overdose mortality increased from 0.8 per 100 000 inhabitants in 2013 to 1.2 per 100 000 inhabitants in 2017.

**Meaning:**

Opioid prescription and associated adverse events are increasing in the Netherlands.

## Teaser

This cohort study uses nationwide data to determine opioid prescription patterns and survey data to examine risk factors associated with opioid prescription in the Netherlands.

## Introduction

Opioids have been used for medicinal, recreational, and religious purposes for more than 5000 years.^1^ In modern medicine, opioids are still the cornerstone of pain pharmacotherapy, eg, in the management of postoperative acute pain and chronic pain associated with cancer.^2,3,4^ An increasing body of evidence shows an increase in the prescription rate for opioids in the treatment of chronic pain not associated with cancer. This puts a large number of individuals at risk for the potentially life-threatening adverse effects of opioids.^5,6^

In the United States, a rapid increase in opioid use has been observed,^7^ and the proportion of US residents who filled at least 1 opioid prescription increased from 4.9% in 2006 to 17.4% in 2017.^8,9^ In 2015, approximately 2 million people in the United States experienced prescription opioid use disorder (ie, addiction),^10^ and there were approximately 400 000 opioid overdose deaths between 1999 and 2017.^11^

In 2015, about half a million residents of the Netherlands (3% of the population) received at least 1 oxycodone or fentanyl prescription, a 67% increase (approximately 200 000 individuals) compared with 2012.^12^ Information on the dynamics of opioid prescription in the Netherlands is needed to prevent an increase in opioid overdose mortalities. Therefore, the aims of this study were to identify opioid prescription patterns and changes in hospital admissions for opioid overdose and opioid overdose mortality in the Netherlands and to examine risk factors associated with opioid prescription in a large repeated national health survey.

## Methods

The institutional review board of the anesthesiology department and intensive care unit of the Leiden University Medical Center approved the study and waived participant consent because we used deidentified data. Analyses were conducted between December 2018 and February 2019. We analyzed several anonymized databases from Statistics Netherlands covering the total population of the Netherlands. Statistics Netherlands collects information from several databases on prescription reimbursement data, hospital admission data with diagnosis, and mortality data on all causes of death and allows linkage on an individual level.

To identify opioid prescriptions, hospital admissions for opioid overdose, and opioid overdose mortalities, we performed an analysis including all individuals who lived in the Netherlands between January 1, 2013, and December 31, 2017. To identify risk factors associated with opioid prescriptions, and in all other analyses, we included participants of the September to November 2012^20^ and the September to November 2016^21^ Dutch Health Monitor (DHM) surveys.

### Opioid Prescription in the Total Dutch Population Through Time

Opioid reimbursement data were collected for all residents of the Netherlands registered in the municipal population register and entitled to pharmaceutical care (ie, basic health insurance). These data come from the Health Care Insurance Board.^13^ Opioids prescribed in hospitals and dispensed from outpatient or community pharmacies or in care homes are collected in national reimbursement data, but medicines dispensed in hospitals are not. Opioids were classified according to the World Health Organization Anatomical Therapeutic Chemical (ATC) classification code N02A.^14^

### Hospital Admissions for Opioid Overdose and Opioid Overdose Deaths in the Total Dutch Population

The Dutch Hospital Data,^15^ a nationwide register of all inpatient hospital admissions and all hospital specialist outpatient clinic and emergency department visits since 2013, contains information about hospital admissions for opioid overdose. Each record contains the date of hospital inpatient and outpatient encounters, the discharge date, and discharge diagnoses. Diagnoses are coded according to the *International Statistical Classification of Diseases, Tenth Revision, Clinical Modification (ICD-10-CM)*.^16^ Hospital admissions for opioid overdose were identified with the F11 and T40 code series.^17^ The Dutch Register of Causes of Death records all deaths in the Netherlands based on death certificates,^18^ encoded according to the *ICD-10-CM*.^16^ Opioid overdose deaths were classified with codes *X40-45*, *Y10-15*, *Y47*, and *Y49*.^19^

### Characteristics of All Participants and of Individuals Who Received Opioid Prescriptions in the DHM

The DHM is a national health survey on well-being at a detailed subpopulation level.^20,21^ The questionnaire was sent out by all Municipal Health Services to different random subsets of the population from September to November 2012 and from September to November 2016. Residents of the Netherlands aged 19 years or older were approached.^20,21^

The DHM is a self-reported survey that includes educational level; marital status; lifestyle habits; employment; self-perceived physical health; ability to meet financial needs; body mass index (BMI), calculated as weight in kilograms divided by height in meters squared; frequency of feelings of depression; and severity of feelings of loneliness (based on an 11-point scale by de Jong-Gierveld et al^22^). Chronic disorders (eg, history of cancer, headache or migraine, neck or shoulder pain, back pain, osteoarthritis of the hip or knee, and rheumatoid arthritis or fibromyalgia) were only queried in the 2012 DHM. Age, sex, immigration status (ie, native, first generation, or second generation [based on the birth certificate]), and income data were obtained by Statistics Netherlands from the municipal registers, and household income data (based on quintile level) were obtained through tax records. We linked the 2012 DHM and 2016 DHM data with national prescription reimbursement data.

### Risk Factors Associated With Opioid Prescription Among Participants in the DHM

Individuals surveyed in the 2012 and 2016 DHMs were considered opioid exposed if they filled at least 1 opioid prescription in that year. Opioid prescriptions were compared between the 2016 and 2012 DHMs.

The DHM oversampled individuals older than 65 years, which hampers direct translation to the total population. Therefore, we estimated age-specific risk rates of opioid prescription based on the age distribution of the Dutch population in a sensitivity analysis.

We investigated the characteristics of individuals who had received 0, 1, 2 to 4, or 5 or more opioid prescriptions in the past 12 months in the 2012 DHM survey. Detailed information about the number of opioid prescriptions was available for the years 2012 through 2016 but not yet for 2017. One opioid prescription was considered a proxy for acute pain, and 5 or more opioid prescriptions was considered a proxy for chronic pain.

### Trajectories of Individuals With Opioid Prescription in the 2012 DHM

We followed individuals who received an opioid prescription in the 2012 DHM for subsequent prescriptions, excluding those who died before January 1, 2013. By linking to the national reimbursement database, we assessed whether patients who had received an opioid prescription in 2012 filled at least 1 opioid prescription from 2013 to 2016, including prescription renewals (>1 opioid prescription). We stratified participants of the 2012 DHM by self-reported morbidity (ie, having cancer or having chronic noncancer pain in 12 months prior to the survey) for the longitudinal analysis of opioid prescription (adjusted for mortality) by linking national prescription and mortality databases.

### Statistical Analysis

To identify opioid prescription rate, hospital admissions for opioid overdose, and opioid overdose deaths, we obtained descriptive statistics for all residents living in the Netherlands between January 1, 2013, to December 31, 2017. Opioid prescriptions are shown as absolute numbers and as a proportion of the total population per calendar year. Findings are also presented as relative risks (RRs) with 95% CIs that were calculated under the Poisson distribution assumption. Hospital admissions for opioid overdose and opioid overdose deaths are presented as numbers per 100 000 inhabitants per calendar year and as RRs with 95% CIs compared with 2013, the reference year. Similar descriptive analyses were performed in the 2012 and 2016 DHMs. To highlight risk factors associated with opioid prescription, we performed logistic regression analysis in the 2012 DHM. In the comparison of the 2016 DHM with the 2012 DHM, findings were adjusted for age, sex, level of education, standardized household income, and marital status by logistic regression. Results are presented as odds ratios (ORs) with 95% CIs. We also performed a longitudinal analysis for opioid prescription from 2013 onward, stratified by self-reported cancer and chronic noncancer pain.

Individuals with missing data for the relevant variables in the 2012 and 2016 DHMs were excluded from the analysis. For total population characteristics, there were no missing data, nor were individuals lost to follow-up. All statistical analyses were performed with SPSS Statistics version 24.0 (IBM).

## Results

### Opioid Prescription, Hospital Admissions for Opioid Overdose, and Opioid Overdose Deaths Among the Total Dutch Population

Among the total population of 16 779 575 people the Netherlands in 2013, 814 211 individuals (4.9%) received an opioid prescription. The number of opioid prescriptions increased to 1 027 019 of 17 081 507 people (6.0%) in 2017, a 24% relative increase. The mean (SD) age of individuals who received an opioid prescription was 59.3 (18.5) years in 2017; 613 203 women (59.7%) and 413 816 men (40.3%) received an opioid prescription (Table 1).

**Table 1.** Opioid Prescription Changes, Hospital Admissions for Opioid Overdose, and Opioid Overdose Deaths in the Netherlands From 2013 to 2017

Outcome	2013 (n = 16 779 575)		2014 (n = 16 829 290)		2015 (n = 16 900 726)		2016 (n = 16 979 120)		2017 (n = 17 081 507)	
	No. (%)	RR (95% CI)	No. (%)	RR (95% CI)	No. (%)	RR (95% CI)	No. (%)	RR (95% CI)	No. (%)	RR (95% CI)
Opioid prescription^a^	814 211 (4.9)	1 [Reference]	863 110 (5.1)	1.06 (1.05-1.06)	921 754 (5.5)	1.12 (1.12-1.13)	975 979 (5.8)	1.18 (1.18-1.19)	1 027 019 (6.0)	1.24 (1.24-1.24)
Hospital admissions for opioid overdose, per 100 000^b^	1537 (9.2)	1 [Reference]	1619 (9.6)	1.05 (0.98-1.13)	1801 (10.7)	1.16 (1.09-1.25)	2115 (12.5)	1.36 (1.27-1.45)	2236 (13.1)	1.43 (1.34-1.52)
Opioid overdose deaths, per 100 000^c^	139 (0.8)	1 [Reference]	139 (0.8)	1.00 (0.79-1.26)	158 (0.9)	1.13 (0.90-1.42)	187 (1.1)	1.33 (1.07-1.66)	211 (1.2)	1.49 (1.20-1.85)

Abbreviation: RR, relative risk.

^a^ Identified by World Health Organization Anatomical Therapeutic Chemical classification code N02A.

^b^ Identified by *International Statistical Classification of Diseases, Tenth Revision, Clinical Modification* (*ICD-10-CM*) codes F11 series and T40 series.

^c^ Identified by *ICD-10-CM* codes X40-45, Y10-15, Y47, and Y49.

We identified an increase in hospital admissions for opioid overdose. This rate was 9.2 per 100 000 inhabitants in 2013 and 13.1 per 100 000 inhabitants in 2017 (RR, 1.43 [95% CI, 1.34-1.52]). Similarly, an increased risk of opioid overdose death was observed, from 0.83 per 100 000 inhabitants in 2013 to 1.2 per 100 000 inhabitants in 2017 (RR, 1.49 [95% CI, 1.20-1.85]) (Table 1).

### Characteristics of All Participants and of Individuals Who Received Opioid Prescriptions in the 2012 and 2016 DHMs

The response rate of the 2012 DHM varied between 45% and 50% per Municipal Health Service. In the 2016 DHM, more people were contacted, but at 40%, the response rate was slightly lower than in 2012. The 2012 DHM contained records for 387 195 individuals (mean [SD] age, 57.27 [17.98] years; 211 281 [54.6%] women), and the 2016 DHM contained records for 457 153 individuals (mean [SD] age, 60.32 [17.25] years; 247 116 [54.1%] women). There was an overlap of 17 502 individuals (2.1%) between 2012 and 2016 DHMs, whereas 826 846 individuals (97.9%) were unique.

Characteristics of participants in the 2012 and 2016 DHM surveys are presented in Table 2. In the 2012 DHM, 29 553 individuals (7.6%) received an opioid prescription (mean [SD] age, 64.8 [15.6] years; 8546 [8.8%] women). Of these, 16 140 (54.6%) received more than 1 opioid prescription (eTable 1 in the Supplement). In the 2016 DHM, 37 458 individuals (8.2%) received an opioid prescription (mean [SD] age, 67.1 [14.4] years; 22 864 [9.3%] women).

**Table 2.** Characteristics of All Participants and of Individuals Who Received Opioid Prescriptions in the 2012 and 2016 Dutch Health Monitor Surveys

Characteristic	No./Total No. (%)			
	2012		2016	
	Overall	Received N02A Prescription	Overall	Received N02A Prescription
Total	387 195 (100)	29 553/387 195 (7.6)	457 153 (100)	37 458/457 153 (8.2)
Sex				
Men	175 914/387 195 (45.4)	11 007/175 914 (6.3)	210 037/457 153 (45.9)	14 594/210 037 (6.9)
Women	211 281/387 195 (54.6)	18 546/211 281 (8.8)	247 116/457 153 (54.1)	22 864/247 116 (9.3)
Age group, y				
19 to 35	53 519/376 384 (14.2)	1492/53 519 (2.8)	50 097/457 153 (11.1)	1370/50 097 (2.7)
>35 to 45	43 166/376 384 (11.5)	1957/43 166 (4.5)	40 134/457 153 (8.8)	1753/40 134 (4.4)
>45 to 55	56 275/376 384 (15.0)	3456/56 275 (6.1)	60 105/457 153 (13.1)	3679/60 105 (6.1)
>55 to 65	60 617/376 384 (16.1)	4480/60 617 (7.4)	71 548/457 153 (15.7)	5510/71 548 (7.7)
>65	162 807/376 384 (43.3)	17 485/162 807 (10.7)	235 269/457 153 (51.5)	25 146/235 269 (10.7)
Highest level of education				
Primary school	37 138/373 998 (9.9)	5080/37 138 (13.7)	31 823/425 731 (7.5)	4602/31 823 (14.5)
High school underclassman^a^	13 1079/373 998 (35.0)	12 387/13 1079 (9.5)	141 231/425 731 (33.2)	14 849/141 231 (10.5)
High school upperclassman^b^	105 863/373 998 (28.3)	6432/105 863 (6.1)	130 099/425 731 (30.6)	9056/130 099 (7.0)
College or more	99 918/373 998 (26.7)	4196/99 918 (4.2)	122 578/425 731 (28.8)	5825/122 578 (4.8)
Immigration status				
Native	335 103/387 195 (86.5)	25 181/335 103 (7.5)	397 808/457 153 (87.0)	32 472/397 808 (8.2)
First generation	28 163/387 195 (7.3)	2457/28 163 (8.7)	32 101/457 153 (7.0)	2674/32 101 (8.3)
Second generation	23 927/387 195 (6.2)	1914/23 927 (8.0)	27 244/457 153 (6.0)	2312/27 244 (8.5)
Standardized household income, quintile^c^				
First	39 072/384 843 (10.2)	3659/39 072 (9.4)	37 996/454 276 (8.4)	3832/37 996 (10.1)
Second	74 437/384 843 (19.3)	8127/74 437 (10.9)	90 475/454 276 (19.9)	11 142/90 475 (12.3)
Third	82 154/384 843 (21.3)	6653/82 154 (8.1)	100 440/454 276 (22.1)	8542/100 440 (8.5)
Fourth	91 375/384 843 (23.7)	5916/91 375 (6.5)	109 165/454 276 (24.0)	7439/109 165 (6.8)
Fifth	97 805/384 843 (25.4)	5119/97 805 (5.2)	116 200/454 276 (25.6)	6385/116 200 (5.5)
Marital status				
Married or in partnership	262 953/370 390 (71.0)	18 655/262 953 (7.1)	316 264/445 961 (70.9)	24 162/316 264 (7.6)
Unmarried or single	42 944/370 390 (11.6)	1858/42 944 (4.3)	46 600/445 961 (10.4)	2179/46 600 (4.7)
Divorced	23 424/370 390 (6.3)	2389/23 424 (10.2)	30 862/445 961 (6.9)	3177/30 862 (10.3)
Widowed	41 069/370 390 (11.1)	5334/41 069 (13.0)	52 235/445 961 (11.7)	6942/52 235 (13.3)
Smoking status				
Nonsmoker	146 773/363 454 (40.4)	9152/146 773 (6.2)	172 754/424 548 (40.7)	11 452/172 754 (6.6)
Former smoker	144 863/363 454 (39.9)	12 007/144 863 (8.3)	182 754/424 548 (43.0)	16 538/182 754 (9.0)
Current smoker	71 818/363 454 (19.8)	6058/71 818 (8.4)	69 040/424 548 (16.3)	6443/69 040 (9.3)
Comorbidity during the past 12 mo				
Cancer	11 026/369 384 (3.0)	2108/11 026 (19.1)	NR	NR
Headache or migraine	47 634/334 901 (14.2)	4940/47 634 (10.4)	NR	NR
Neck or shoulder pain	39 242/337 196 (11.6)	6819/39 242 (17.4)	NR	NR
Back pain	42 699/337 289 (12.7)	9163/42 699 (21.5)	NR	NR
Arthrosis of the hip or knee	72 142/338 617 (21.3)	11 895/72 142 (16.5)	NR	NR
Rheumatoid arthritis or fibromyalgia	24 761/336 003 (7.4)	5274/24 761 (21.3)	NR	NR
Feelings of depression				
Always	2404/365 277 (0.7)	467/2404 (19.4)	2943/436 725 (0.7)	627/2943 (21.3)
Often	8979/365 277 (2.5)	1442/8979 (16.1)	10 872/436 725 (2.5)	1864/10 872 (17.1)
Sometimes	36 730/365 277 (10.1)	4368/36 730 (11.9)	46 831/436 725 (10.7)	6186/46 831 (13.2)
Rarely	89 587/365 277 (24.5)	7591/89 587 (8.5)	116 461/436 725 (26.7)	10 221/116 461 (8.8)
Never	227 577/365 277 (62.3)	13 666/227 577 (6.0)	259 618/436 725 (59.4)	16 617/259 618 (6.4)
Feeling of loneliness^d^				
Not lonely	216 407/358 213 (60.4)	13 581/216 407 (6.3)	239 971/450 146 (53.3)	16 285/239 971 (6.8)
Somewhat lonely	114 222/358 213 (31.9)	9636/114 222 (8.4)	144 307/450 146 (32.1)	13 026/144 307 (9.0)
Lonely	17 980/358 213 (5.0)	2175/17 980 (12.1)	23 816/450 146 (5.3)	2908/23 816 (12.2)
Very lonely	9604/358 213 (2.7)	1326/9604 (13.8)	12 695/450 146 (2.8)	1754/12 695 (13.8)
Ability to meet financial needs				
No difficulties	160 284/363 596 (44.1)	9615/160 284 (6.0)	217 319/421 510 (51.6)	14 036/217 319 (6.5)
Just able	138 441/363 596 (38.1)	10 718/138 441 (7.7)	147 605/421 510 (35.0)	12 973/147 605 (9.0)
Some difficulties	49 777/363 596 (13.7)	5141/49 777 (10.3)	44 072/421 510 (10.5)	5274/44 072 (12.2)
Great difficulties	15 094/363 596 (4.2)	2129/15 094 (14.1)	12 514/421 510 (3.0)	1955/12 514 (15.6)
Other variables				
Heavy drinker^e^	30 585/357 491 (8.6)	1791/30 585 (5.9)	34 682/423 970 (8.2)	2304/34 682 (6.6)
Live alone	70 210/341 606 (20.6)	7533/70 210 (10.7)	95 136/446 588 (21.3)	10 604/95 136 (11.1)
Unemployed	8369/349 560 (2.4)	636/8369 (7.6)	8571/407 636 (2.1)	625/8571 (7.3)
Physical health				
Very good or good	276 830/382 208 (72.4)	11 265/276 830 (4.1)	323 416/451 423 (71.6)	14 318/323 416 (4.4)
Fair	89 435/382 208 (23.4)	12 776/89 435 (14.3)	107 125/451 423 (23.7)	15 866/107 125 (14.8)
Poor or very poor	15 943/382 208 (4.2)	4880/15 943 (30.6)	20 882/451 423 (4.6)	6698/20 882 (32.1)
BMI				
<18.5	5061/371 808 (1.4)	506/5061 (10.0)	5642/435 892 (1.3)	588/5642 (10.4)
18.5 to 20	13 845/371 808 (3.6)	768/13 845 (5.7)	15 186/435 892 (3.5)	963/15 186 (6.3)
>20 to 25	159 525/371 808 (42.9)	9344/159 525 (5.9)	180 510/435 892 (41.4)	11 105/180 510 (6.2)
>25 to 30	143 098/371 808 (38.5)	11 198/143 098 (7.8)	168 617/435 892 (38.7)	13 788/168 617 (8.2)
>30	50 639/371 808 (13.6)	6120/50 639 (12.1)	65 937/435 892 (15.1)	8662/65 937 (13.1)

Abbreviations: BMI, body mass index (calculated as weight in kilograms divided by height in meters squared); N02A, World Health Organization Anatomical Therapeutic Chemical classification code for an opioid; NR, not reported.

^a^ Junior secondary vocational education (ie, MAVO or LBO in the Dutch educational system).

^b^ Senior secondary general education, university preparatory education, or senior secondary vocational education (ie, HAVO, VWO, or MBO, respectively, in the Dutch educational system).

^c^ First indicates incomes in the lowest 20% and fifth indicates incomes in the highest 20% of all reported incomes.

^d^ Measured using the scale by de Jong-Gierveld et al.^22^

^e^ Defined as at least 4 units of alcohol for women or 6 units of alcohol for men per day at least once per week.

### Risk Factors Associated With Opioid Prescription

Risk factors associated with opioid prescription are presented in Table 3. Among others, we found that being older than 65 years (OR, 4.20 [95% CI, 3.98-4.43]), being a woman (OR, 1.44 [95% CI, 1.41-1.48]), having only completed primary school (OR, 3.62 [95% CI, 3.46-3.77]), being divorced (OR, 2.51 [95% CI, 2.36-2.67]) or widowed (OR, 3.30 [95% CI, 3.13-3.49]), smoking (OR, 1.39 [95% CI, 1.34-1.43]), or having a BMI greater than 30 (OR, 2.28 [95% CI, 2.11-2.46]) were associated with opioid prescription in the 2012 DHM. Other individual characteristics associated with opioid prescription were back pain (OR, 4.34 [95% CI, 4.23-4.46]), rheumatoid arthritis or fibromyalgia (OR, 3.77 [95% CI, 3.65-3.90]), or cancer (OR, 3.00 [95% CI, 2.86-3.15]). Factors related to emotional well-being and stress, such as increased frequency of feelings of depression (always vs never: OR, 3.77 [95% CI, 3.41-4.18]), increased severity of feelings of loneliness (very lonely vs not lonely: OR, 2.39 [95% CI, 2.25-2.54]), and having difficulty meeting financial needs (great difficulties vs no difficulties: OR, 2.57 [95% CI, 2.45-2.71]) were also associated with increased risk of opioid prescription with increasing severity. Individuals who reported poor physical health status had increased odds of opioid prescription compared with individuals who reported good physical health (OR, 10.40 [95% CI, 10.01-10.81]). We did not find an association of opioid prescription with being unemployed (OR, 1.05 [95% CI, 0.96-1.13]), and unhealthy alcohol use, defined as at least 4 units of alcohol for women or 6 units of alcohol for men per day at least once per week, was negatively associated with opioid prescription (OR, 0.76 [95% CI, 0.73-0.80]). Opioids were prescribed more than once during the year for more than 50% of individuals who received a prescription, with the highest number of repeated prescriptions in groups in which we also found the highest ORs (eTable 1 in the Supplement).

**Table 3.** Risk Factors Associated With Opioid Prescription in the 2012 Dutch Health Monitor Survey and Increase in Opioid Prescription in the 2016 Dutch Health Monitor Survey

Factor	Odds Ratio (95% CI)	
	2012 N02A Prescription	2016 vs 2012 N02A Prescription, Adjusted^a^
Total	1 [Reference]	1.05 (1.03-1.06)
Sex		
Men	1 [Reference]	1.06 (1.03-1.09)
Women	1.44 (1.41-1.48)	1.04 (1.02-1.06)
Age group, y		
19 to 35	1 [Reference]	1.05 (0.97-1.14)
>35 to 45	1.66 (1.55-1.77)	1.04 (0.97-1.11)
>45 to 55	2.28 (2.15-2.43)	1.05 (1.00-1.11)
>55 to 65	2.78 (2.62-2.95)	1.08 (1.03-1.13)
>65	4.20 (3.98-4.43)	1.04 (1.01-1.06)
Highest level of education		
Primary school	3.62 (3.46-3.77)	1.06 (1.01-1.11)
High school underclassman^b^	2.38 (2.30-2.47)	1.06 (1.04-1.09)
High school upperclassman^c^	1.48 (1.42-1.54)	1.04 (1.00-1.07)
College or more	1 [Reference]	1.01 (0.97-1.06)
Immigration status		
Native	1 [Reference]	1.06 (1.04-1.08)
First generation	1.18 (1.13-1.23)	0.94 (0.88-1.00)
Second generation	1.07 (1.02-1.12)	1.01 (0.95-1.08)
Standardized household income, quintile^d^		
First	1.87 (1.79-1.96)	1.07 (1.02-1.13)
Second	2.22 (2.14-2.30)	1.11 (1.07-1.14)
Third	1.60 (1.54-1.66)	1.00 (0.97-1.04)
Fourth	1.25 (1.21-1.30)	1.01 (0.97-1.05)
Fifth	1 [Reference]	1.02 (0.98-1.06)
Marital status		
Married or in partnership	1.69 (1.61-1.77)	1.05 (1.03-1.07)
Unmarried or single	1 [Reference]	1.02 (0.95-1.09)
Divorced	2.51 (2.36-2.67)	1.00 (0.94-1.06)
Widowed	3.30 (3.13-3.49)	1.06 (1.01-1.10)
Smoking status		
Nonsmoker	1 [Reference]	1.03 (0.99-1.06)
Former smoker	1.36 (1.32-1.40)	1.07 (1.04-1.09)
Current smoker	1.39 (1.34-1.43)	1.07 (1.03-1.12)
Comorbidity during the past 12 mo^e^		
Cancer	3.00 (2.86-3.15)	NR
Headache or migraine	1.48 (1.43-1.53)	NR
Neck or shoulder pain	3.00 (2.92-3.10)	NR
Back pain	4.34 (4.23-4.46)	NR
Arthrosis of the hip or knee	3.33 (3.24-3.41)	NR
Rheumatoid arthritis or fibromyalgia	3.77 (3.65-3.90)	NR
Feelings of depression		
Always	3.77 (3.41-4.18)	1.09 (0.94-1.25)
Often	3.00 (2.82-3.18)	1.06 (0.97-1.14)
Sometimes	2.11 (2.04-2.19)	1.08 (1.04-1.13)
Rarely	1.45 (1.41-1.49)	1.01 (0.97-1.04)
Never	1 [Reference]	1.02 (0.99-1.05)
Feeling of loneliness^f^		
Not lonely	1 [Reference]	1.04 (1.02-1.07)
Somewhat lonely	1.38 (1.34-1.41)	1.04 (1.01-1.07)
Lonely	2.06 (1.96-2.16)	0.99 (0.93-1.06)
Very lonely	2.39 (2.25-2.54)	1.01 (0.93-1.09)
Ability to meet financial needs		
No difficulties	1 [Reference]	1.05 (1.02-1.08)
Just able	1.32 (1.28-1.35)	1.09 (1.06-1.12)
Some difficulties	1.81 (1.74-1.87)	1.09 (1.04-1.14)
Great difficulties	2.57 (2.45-2.71)	1.09 (1.02-1.17)
Other factors^g^		
Heavy drinker^h^	0.76 (0.73-0.80)	1.04 (0.98-1.12)
Live alone	1.69 (1.64-1.74)	1.03 (1.00-1.06)
Unemployed	1.05 (0.96-1.13)	0.96 (0.85-1.08)
Physical health		
Very good or good	1 [Reference]	1.04 (1.01-1.07)
Fair	3.93 (3.83-4.04)	1.03 (1.00-1.06)
Poor or very poor	10.40 (10.01-10.81)	1.08 (1.03-1.13)
BMI		
<18.5	1.84 (1.64-2.07)	0.93 (0.81-1.07)
18.5 to 20	1 [Reference]	1.02 (0.92-1.07)
>20 to 25	1.03 (0.96-1.11)	1.01 (0.98-1.04)
>25 to 30	1.41 (1.30-1.52)	1.03 (0.99-1.05)
>30	2.28 (2.11-2.46)	1.09 (1.05-1.13)

Abbreviations: BMI, body mass index (calculated as weight in kilograms divided by height in meters squared); N02A, World Health Organization Anatomical Therapeutic Chemical classification code for an opioid; NR, not reported.

^a^ Adjusted for age, sex, level of education, standardized household income, and marital status.

^b^ Junior secondary vocational education (ie, MAVO or LBO in the Dutch educational system).

^c^ Senior secondary general education, university preparatory education, or senior secondary vocational education (HAVO, VWO, or MBO, respectively, in the Dutch educational system).

^d^ First indicates incomes in the lowest 20% of all reported incomes, and fifth indicates incomes in the highest 20%.

^e^ Compared with not having any of the listed comorbidities.

^f^ Measured using the scale by de Jong-Gierveld et al.^22^

^g^ Compared with nonheavy drinker, not living alone, and employed.

^h^ Defined as at least 4 units of alcohol for women or 6 units of alcohol for men per day at least once per week.

Opioid prescriptions increased between the 2012 DHM and the 2016 DHM (adjusted OR, 1.05 [95% CI, 1.03-1.06]). Risk factors associated with opioid prescription that were identified in the 2012 DHM were also associated with opioid prescription in the 2016 DHM. Furthermore, groups that were not associated with increased risk of opioid prescription in the 2012 DHM were more likely to receive an opioid prescription in the 2016 DHM, as most ORs were higher than 1.00. Opioid prescription rates in the 2012 and 2016 DHM surveys were similar to those of the whole country when stratified by age (eTable 2 in the Supplement).

### Risk Factors for Single and Multiple Opioid Prescriptions Among Participants in the 2012 DHM

We classified participants according to the number of prescriptions they filled in the 2012 DHM. Among 29 553 individuals who filled at least 1 opioid prescription in the 2012 DHM, 13 413 (45.4%) filled only 1 opioid prescription, 8928 (30.2%) filled 2 to 4 prescriptions, and 7212 (24.4%) filled 5 or more opioid prescriptions (eTable 3 in the Supplement). Risk factors associated with 1, 2 to 4, and 5 or more opioid prescriptions are presented in Table 4 (crude numbers are presented in eTable 4 in the Supplement). Repeated prescriptions were associated with previously identified risk factors for opioid prescription, and 5 or more prescriptions were particularly associated with these same risk factors, such as being older than 65 years (OR, 10.89 [95% CI, 9.27-12.79]), having great difficulty meeting financial needs (OR 3.42 [95% CI, 3.13-3.74]), having only a primary school education (OR, 6.26 [95% CI, 5.73-6.84]), being divorced (OR, 2.90 [95% CI, 2.55-3.29) or widowed (OR, 4.68 [95% CI, 4.20-5.22]), and having comorbidities (cancer: OR, 4.22 [95% CI, 3.91-4.56]; back pain: OR, 7.44 [95% CI, 7.10-7.81]; arthrosis of the hip or knee: OR, 4.90 [95% CI, 4.67-5.13]; rheumatoid arthritis or fibromyalgia: OR, 5.55 [95% CI, 5.25-5.85]).

**Table 4.** Risk Factors Associated With Number of Opioid Prescriptions in the 2012 Dutch Health Monitor

Factor	Odds Ratio (95% CI)		
	1 N02A Prescription	2-4 N02A Prescriptions	≥5 N02A Prescriptions
Sex			
Men	1 [Reference]	1 [Reference]	1 [Reference]
Women	1.27 (1.22-1.31)	1.43 (1.37-1.50)	1.72 (1.64-1.81)
Age group, y			
19 to 35	1 [Reference]	1 [Reference]	1 [Reference]
>35 to 45	1.47 (1.35-1.61)	1.80 (1.58-2.06)	2.30 (1.89-2.79)
>45 to 55	1.77 (1.63-1.91)	2.62 (2.32-2.96)	4.27 (3.58-5.09)
>55 to 65	1.97 (1.82-2.12)	3.41 (3.04-3.83)	5.63 (4.75-6.68)
>65	2.43 (2.28-2.60)	5.13 (4.61-5.70)	10.89 (9.27-12.79)
Highest level of education			
Primary school	2.28 (2.14-2.43)	3.70 (3.43-3.99)	6.26 (5.73-6.84)
High school underclassman^a^	1.86 (1.77-1.95)	2.45 (2.30-2.62)	3.41 (3.14-3.70)
High school upperclassman^b^	1.35 (1.28-1.43)	1.45 (1.35-1.56)	1.79 (1.63-1.96)
College or more	1 [Reference]	1 [Reference]	1 [Reference]
Immigration status			
Native	1 [Reference]	1 [Reference]	1 [Reference]
First generation	1.24 (1.17-1.32)	1.25 (1.16-1.34)	0.93 (0.85-1.02)
Second generation	1.08 (1.00-1.15)	1.03 (0.95-1.13)	1.09 (0.99-1.20)
Standardized household income, quintile^c^			
First	1.42 (1.33-1.52)	1.88 (1.74-2.03)	2.91 (2.66-3.18)
Second	1.53 (1.45-1.61)	2.25 (2.11-2.40)	3.75 (3.47-4.05)
Third	1.36 (1.30-1.44)	1.58 (1.47-1.69)	2.14 (1.97-2.33)
Fourth	1.15 (1.09-1.21)	1.25 (1.17-1.34)	1.51 (1.38-1.65)
Fifth	1 [Reference]	1 [Reference]	1 [Reference]
Marital status			
Married or in partnership	1.54 (1.44-1.65)	1.86 (1.70-2.04)	1.68 (1.52-1.87)
Unmarried or single	1 [Reference]	1 [Reference]	1 [Reference]
Divorced	1.95 (1.79-2.14)	2.88 (2.57-3.23)	2.90 (2.55-3.29)
Widowed	2.18 (2.02-2.36)	3.56 (3.22-3.94)	4.68 (4.20-5.22)
Smoking			
Nonsmoker	1 [Reference]	1 [Reference]	1 [Reference]
Former smoker	1.26 (1.21-1.31)	1.40 (1.34-1.48)	1.42 (1.34-1.50)
Current smoker	1.25 (1.19-1.32)	1.38 (1.30-1.47)	1.57 (1.47-1.67)
Comorbidity during the past 12 mo			
Cancer	2.03 (1.88-2.20)	2.64 (2.43-2.87)	4.22 (3.91-4.56)
Headache or migraine	1.39 (1.32-1.45)	1.42 (1.34-1.50)	1.60 (1.51-1.70)
Neck or shoulder pain	2.12 (2.02-2.21)	2.92 (2.78-3.07)	3.94 (3.74-4.15)
Back pain	2.31 (2.21-2.41)	4.17 (4.00-4.37)	7.44 (7.10-7.81)
Arthrosis of the hip or knee	2.23 (2.15-2.31)	3.25 (3.12-3.39)	4.90 (4.67-5.13)
Rheumatoid arthritis or fibromyalgia	2.31 (2.20-2.43)	3.40 (3.22-3.60)	5.55 (5.25-5.85)
Feelings of depression			
Always	2.04 (1.72-2.42)	3.58 (3.02-4.24)	6.69 (5.70-7.84)
Often	1.89 (1.72-2.07)	2.77 (2.50-3.07)	5.10 (4.62-5.62)
Sometimes	1.54 (1.46-1.62)	2.05 (1.93-2.18)	3.25 (3.04-3.47)
Rarely	1.22 (1.17-1.27)	1.44 (1.36-1.51)	1.95 (1.84-2.06)
Never	1 [Reference]	1 [Reference]	1 [Reference]
Feeling of loneliness^d^			
Not lonely	1 [Reference]	1 [Reference]	1 [Reference]
Somewhat lonely	1.17 (1.13-1.22)	1.44 (1.38-1.52)	1.66 (1.57-1.75)
Lonely	1.51 (1.41-1.63)	2.11 (1.95-2.30)	2.84 (2.60-3.10)
Very lonely	1.68 (1.53-1.84)	2.17 (1.95-2.42)	3.78 (3.42-4.18)
Ability to meet financial needs			
No difficulties	1 [Reference]	1 [Reference]	1 [Reference]
Just able	1.21 (1.16-1.26)	1.35 (1.29-1.42)	1.43 (1.35-1.52)
Some difficulties	1.54 (1.46-1.62)	1.78 (1.67-1.89)	2.18 (2.04-2.34)
Great difficulties	1.90 (1.76-2.05)	2.53 (2.32-2.76)	3.42 (3.13-3.74)
Other factors^e^			
Heavy drinker^f^	0.87 (0.81-0.93)	0.82 (0.75-0.89)	0.53 (0.47-0.60)
Live alone	1.27 (1.22-1.33)	1.72 (1.64-1.81)	2.36 (2.24-2.48)
Unemployed	1.12 (1.00-1.25)	1.15 (1.00-1.32)	0.77 (0.64-0.93)
Physical health			
Very good or good	1 [Reference]	1 [Reference]	1 [Reference]
Fair	2.45 (2.36-2.54)	4.07 (3.88-4.27)	8.71 (8.17-9.29)
Poor or very poor	3.41 (3.20-3.63)	8.51 (7.98-9.07)	33.43 (31.15-35.88)
BMI			
<18.5	1.41 (1.18-1.69)	1.97 (1.58-2.45)	2.20 (1.80-2.68)
18.5 to 20	1 [Reference]	1 [Reference]	1 [Reference]
>20 to 25	1.08 (0.97-1.21)	1.17 (1.01-1.35)	0.82 (0.72-0.95)
>25 to 30	1.42 (1.28-1.59)	1.69 (1.46-1.95)	1.06 (0.93-1.22)
>30	1.99 (1.77-2.23)	2.77 (2.39-3.21)	1.94 (1.68-2.23)

Abbreviations: BMI, body mass index (calculated as weight in kilograms divided by height in meters squared); N02A, World Health Organization Anatomical Therapeutic Chemical classification code for an opioid.

^a^ Junior secondary vocational education (ie, MAVO or LBO in the Dutch educational system).

^b^ Senior secondary general education, university preparatory education, or senior secondary vocational education (HAVO, VWO, or MBO, respectively, in the Dutch educational system).

^c^ First indicates incomes in the lowest 20% of all reported incomes, and fifth indicates incomes in the highest 20%.

^d^ Measured using the scale by de Jong-Gierveld et al.^22^

^e^ Compared with nonheavy drinker, not living alone, and employed.

^f^ Defined as at least 4 units of alcohol for women or 6 units of alcohol for men per day at least once per week.

### Trajectories of Individuals With Opioid Prescriptions in the 2012 DHM

We followed participants of the 2012 DHM by linking them to national prescription statistics (Table 5). Of 386 470 participants who responded to 2012 DHM, 29 553 participants (7.6%) received an opioid prescription, which increased to 33 626 participants (9.1%) in 2016. Opioid prescription prevalence increased from 7.6% in 2012 to 12.2% in 2013 and to 22.7% in 2016, meaning that 22.7% of participants in the 2012 DHM had received an opioid prescription during the 4-year follow-up. We stratified participants in the 2012 DHM by cancer pain vs noncancer pain history. Of 11 026 participants who reported having cancer in 2012, 2108 (19.1%) received an opioid prescription in 2012. By 2013, 2054 individuals with cancer pain (19.0%) had received an opioid prescription in the past year, and this rate further decreased to 1531 individuals (17.6%) in 2016 (RR, 0.93 [95% CI, 0.87-0.99]). In 2013, 17 481 of 143 357 participants with noncancer pain (12.2%) reimbursed an opioid prescription, and by 2016, 19 233 of 136 674 participants with noncancer pain (14.1%) had reimbursed an opioid prescription (RR, 1.15 [95% CI, 1.13-1.18]). In 2016, opioid prescription prevalence at the 4-year follow-up was 39.8% (3458 participants) among the group with cancer pain (RR, 1.37 [95% CI, 1.30-1.43]) and 33.5% (44 775 participants) among the group with noncancer pain (RR, 1.71 [95% CI, 1.67-1.72]).

**Table 5.** Trajectories of Individuals With Opioid Prescription in the 2012 Dutch Health Monitor Survey Stratified by Self-reported Cancer or Noncancer Pain^a^

Outcome	No. (%)			
	2013 (n = 386 470)	2014 (n = 381 818)	2015 (n = 376 667)	2016 (n = 370 747)
Opioid prescription incidence, past 12 mo	28 929 (7.5)	30 476 (8.0)	32 405 (8.6)	33 626 (9.1)
Opioid prescription prevalence, past 4 y	47 289 (12.2)	60 841 (15.9)	73 155 (19.4)	84 195 (22.7)
Death since 2012	4652 (1.2)	5151 (1.3)	5920 (1.6)	6432 (1.7)
Cancer pain	10 830 (2.8)	9880 (2.6)	9202 (2.4)	8681 (2.3)
Opioid prescription incidence, past 12 mo	2054 (19.0)	1701 (17.2)	1615 (17.6)	1531 (17.6)
RR (95% CI)	1 [Reference]	0.91 (0.85-0.97)	0.93 (0.87-0.99)	0.93 (0.87-0.99)
1 Opioid prescription	524 (4.8)	484 (4.9)	489 (5.3)	444 (5.1)
>1 Opioid prescription	1530 (14.1)	1217 (12.3)	1126 (12.2)	1087 (12.5)
Opioid prescription prevalence, past 4 y	3157 (29.2)	3163 (32.0)	3300 (35.9)	3458 (39.8)
RR (95% CI)	1 [Reference]	1.10 (1.05-1.15)	1.23 (1.17-1.29)	1.37 (1.30-1.43)
Death since 2012	950 (8.8)	678 (6.9)	521 (5.7)	490 (5.6)
Noncancer pain^b^	143 357 (37.1)	141 348 (37.0)	139 165 (36.9)	136 674 (36.9)
Opioid prescription incidence, past 12 mo	17 481 (12.2)	17 895 (12.7)	18 766 (13.5)	19 233 (14.1)
RR (95% CI)	1 [Reference]	1.04 (1.02-1.06)	1.11 (1.08-1.13)	1.15 (1.13-1.18)
1 Opioid prescription	6785 (4.7)	6514 (4.6)	6608 (4.7)	6528 (4.8)
>1 Opioid prescription	10 696 (7.5)	11 381 (8.1)	12 158 (8.7)	12 705 (9.3)
Opioid prescription prevalence, past 4 y	28 259 (19.7)	34 958 (24.7)	40 838 (29.3)	45 775 (33.5)
RR (95% CI)	1 [Reference]	1.25 (1.24-1.27)	1.49 (1.47-1.51)	1.71 (1.67-1.72)
Deaths since 2012	2009 (1.4)	2183 (1.5)	2491 (1.8)	2787 (2.0)

Abbreviation: RR, relative risk.

^a^ Of 387 195 individuals enrolled in the 2012 Dutch Health Monitor survey, 725 (0.2%) died before January 1, 2013. Percentages shown are cumulative incidences of those who were alive on January 1 per calendar year unless otherwise specified.

^b^ Noncancer pain was identified as self-reported headache or migraine, neck or shoulder pain, back pain, arthrosis of hip or knee, or rheumatoid arthritis or fibromyalgia.

## Discussion

In this study using data with national coverage, we found an increase in opioid prescriptions in the Netherlands from 2012 to 2017. In addition, hospital admissions for opioid overdose and opioid overdose mortality increased during this same period. Several risk factors associated with opioid prescription were identified from 2 large national health surveys in 2012 and 2016.

The increase in opioid prescription, from 4.9% to 6.0% overall, occurred in all age groups, including opioid-naive individuals. This finding was consistent with results from the Dutch Foundation for Pharmaceutical Statistics,^12^ which reported an increase in the number of opioid prescriptions from 2008 to 2016. One of the reasons for the increase may be the introduction of the Dutch National Patient Safety Program, which evaluates hospital performance and patient satisfaction in all hospitals in the Netherlands. One of the benchmarks for hospital performance is postoperative pain and its effective treatment.^23,24^ This may encourage physicians to prescribe more analgesics to combat patient pain scores more effectively. Another important aspect may be the reintroduction of oxycodone in postoperative pain management guidelines in 2013.^25^ Oxycodone has high abuse potential and has been among the drugs most frequently involved in opioid overdoses.^26^ Another possible reason for the increase in opioid prescriptions could be that Dutch physicians may feel that there are no viable alternatives to opioids for the treatment of moderate to severe pain, particularly because of increased awareness of possible gastrointestinal and cardiac adverse effects associated with nonsteroidal anti-inflammatory drugs.^25,27,28^

The increase in hospital admissions for opioid overdose and opioid overdose mortality is a cause for concern. However, these numbers are still considerably lower than those reported in the literature, mainly from the United States.^10,29,30^ There are important differences in the organization of health care between the Netherlands and the United States, and this might account for the difference in morbidity rates. Because of the higher population density (and, concomitantly, health care facility density) in the Netherlands, access to health care is fast and relatively easy. People in need of emergency medical care can generally be reached by emergency medical services within a short time (<15 minutes)^31^ and thus can receive appropriate treatment quickly.

We were unable to identify specific risk factors for hospital admissions for opioid overdose or opioid overdose deaths because of the low absolute number of such incidents. However, the increase in opioid prescription in the Netherlands coincided with opioid-related morbidity and mortality between 2013 and 2017. This suggests that risk factors associated with opioid prescription may also be associated with opioid-related morbidity and mortality.

We identified several risk factors associated with opioid prescription in the Netherlands. To our knowledge, earlier studies^11,30,32^ on risk factors associated with opioid prescription did not include the Dutch population. Previously identified risk factors associated with opioid prescription include older age (>55 years), female sex,^8^ smoking,^34^ alcohol consumption,^33,36^ obesity,^38^ lower socioeconomic status,^37^ unemployment, and depression and anxiety.^35,39^ Most of the risk factors identified in our study were consistent with these, although there were some important differences. We confirmed that female sex, older age, lower socioeconomic status, smoking, and obesity were associated with an increased risk of opioid prescription. Additionally, we observed that poor self-perceived health, depressive symptoms and loneliness, lower household income, and being divorced or widowed were associated with opioid prescription. Other studies have reported an association of alcohol consumption,^40^ ethnicity,^41^ and unemployment^42^ with opioid prescription. We were unable to replicate these findings in our study population.

We found a high number of opioid prescriptions among individuals who self-reported pain symptoms unrelated to cancer. Participants with back pain, rheumatoid arthritis, or fibromyalgia had a similar or even higher prevalence of opioid prescription than participants with cancer pain. During the course of several years, as noted in our longitudinal analysis (Table 5), we saw an increasing prevalence of opioid use among participants who reported noncancer pain, whereas the opioid prescription prevalence among participants with cancer pain remained fairly constant. This suggests a high prescription rate for chronic noncancer pain, despite a lack of evidence for effectiveness of prolonged opioid use.^43,44^ Long-term opioid therapy is associated with increased risk of opioid use disorder or dependence and of occurrence of adverse events, including opioid overdose mortality.^44^

### Limitations

There were several limitations to our study. We did not have individual Anatomical Therapeutic Chemical classification codes for the opioids used, so we were unable to differentiate between strong and weak opioids or direct vs controlled-release preparations, nor could we comment on trends in individual drugs. We identified prolonged opioid use, but we have no data available for the total days of supply and individual doses (and therefore, the morphine milligram equivalents). In addition, the comorbidity survey was only included in the 2012 DHM, so calculations for the risk of opioid prescription through time for these conditions could not be made.

The response rates for the 2012 and 2016 DHMs were not optimal (response rates, 40%-45%), which leads to the question of whether the results can be extrapolated to the total Dutch population. However, since the sampling strategy and population characteristics (mean age and sex distribution) of individuals who were approached in the DHMs were similar, a comparison between the 2 DHMs is valid. Furthermore, when we compared opioid use in the Netherlands stratified by age, results of opioid use in the 2012 and 2016 DHMs were similar to the use of opioids in the whole country as assessed by national data from the Statistics Netherlands, indicating that the low response rates had not biased the results.

Except for the 2012 and 2016 DHMs, all databases used in this study were national statistics data, meaning they contained information about all residents of the Netherlands. However, the DHM is a national survey performed every 4 years that invites approximately 1 million citizens to complete it. Although approximately 40% of approached individuals actually responded to the survey, the total population of respondents is high (approximately 400 000 individuals).

## Conclusions

In conclusion, we found that the number of opioid prescriptions in the Netherlands was increasing. Further research is needed to identify the exact opioids being prescribed and the possible causes for the increase, as well as establishing populations at risk. Currently, the risks of hospital admission for opioid overdose and opioid overdose death are still low, but they are increasing; therefore, prescription of opioids should be monitored closely, and measures should to be taken to prevent a possible opioid epidemic in the Netherlands.

## Notes

Supplement. **eTable 1.** Characteristics of Individuals in the 2012 and 2016 Dutch Health Monitor Surveys Who Received 1 or More Opioid Prescriptions **eTable 2.** Opioid Prescription in the Netherlands Stratified by Age Group **eTable 3.** Number of Individuals in the 2012 and 2016 Dutch Health Monitor Surveys Who Reimbursed 1, 2 to 4, or 5 or More Opioid Prescriptions **eTable 4.** Characteristics of Individuals in the 2012 Dutch Health Monitor Survey Who Reimbursed 0, 1, 2 to 4, or 5 or More Opioid Prescriptions
