# Supplementary material for: Opioid Prescription Patterns and Risk Factors Associated With Opioid Use in the Netherlands
Source: JAMA Netw Open. 2019 Aug 28;2(8):e1910223. doi: 10.1001/jamanetworkopen.2019.10223 (PMC6716286; doi:10.1001/jamanetworkopen.2019.10223)
Supplement: Supplement. — eTable 1. Characteristics of Individuals in the 2012 and 2016 Dutch Health Monitor Surveys Who Received 1 or More Opioid Prescriptions eTable 2. Opioid Prescription in the Netherlands Stratified by Age Group eTable 3. Number of Individuals in the 2012 and 2016 Dutch Health Monitor Surveys Who Reimbursed 1, 2 to 4, or 5 or More Opioid Prescriptions eTable 4. Characteristics of Individuals in the 2012 Dutch Health Monitor Survey Who Reimbursed 0, 1, 2 to 4, or 5 or More Opioid Prescriptions [file jamanetwopen-2-e1910223-s001.pdf]

Supplementary Online Content

Bedene A, Lijfering WM, Niesters M, et al. Opioid prescription patterns and risk factors associated with opioid use in the Netherlands. *JAMA Netw Open*. 2019;2(8):e1910223. doi:10.1001/jamanetworkopen.2019.10223

eTable 1. Characteristics of Individuals in the 2012 and 2016 Dutch Health Monitor Surveys Who Received 1 or More Opioid Prescriptions

eTable 2. Opioid Prescription in the Netherlands Stratified by Age Group

eTable 3. Number of Individuals in the 2012 and 2016 Dutch Health Monitor Surveys Who Reimbursed 1, 2 to 4, or 5 or More Opioid Prescriptions

eTable 4. Characteristics of Individuals in the 2012 Dutch Health Monitor Survey Who Reimbursed 0, 1, 2 to 4, or 5 or More Opioid Prescriptions

This supplementary material has been provided by the authors to give readers additional information about their work.

© 2019 Bedene A et al. *JAMA Network Open.*

eTable 1. Characteristics of Individuals in the 2012 and 2016 Dutch Health Monitor Surveys Who Received 1 or More Opioid Prescriptions

	**2012 Rx, N02A n (%)**	**2016 Rx, N02A n (%)**	**2012 > 1 Rx, N02A n (%)**	**2016 > 1 Rx, N02A n (%)**
Total	29553 (7.6)	37458 (8.2)	16140 (55)	22167 (59)
**Sex**				
Men	11007 (6.3)	14594 (6.9)	5665 (52)	8257 (57)
Women	18546 (8.8)	22864 (9.3)	10475 (57)	13910 (61)
**Age group, years**				
19-35	1492 (2.8)	1370 (2.7)	515 (35)	544 (40)
35-45	1957 (4.5)	1753 (4.4)	806 (41)	831 (47)
45-55	3456 (6.1)	3679 (6.1)	1668 (48)	1962 (53)
55-65	4480 (7.4)	5510 (7.7)	2341 (52)	3115 (57)
> 65	17485 (11)	25146 (11)	10436 (60)	15715 (63)
**Highest level of education**				
Primary school	5080 (14)	4602 (15)	3209 (63)	3062 (67)
High school, underclassman[a]	12387 (10)	14849 (11)	6956 (56)	9158 (62)
High School, upperclassman[b]	6432 (6.1)	9056 (7.0)	3196 (50)	5030 (56)
College or more	4196 (4.2)	5825 (4.8)	1922 (46)	3020(52)
**Immigration status**				
Native (Dutch)	25181 (7.5)	32472 (8.2)	13817 (54)	19260 (59)
First generation	2457 (8.7)	2674 (8.3)	1279 (52)	1506 (56)
Second generation	1914 (8.0)	2312 (8.5)	1043 (55)	1401 (61)
**Standardized household income**				
First quintile	3659 (9.4)	3832 (10)	2131 (58)	2433 (64)
Second quintile	8127 (11)	11142 (12)	5008 (62)	7258 (65)
Third quintile	6653 (8.1)	8542 (8.5)	3566 (54)	5050 (59)
Fourth quintile	5916 (6.5)	7439 (6.8)	3003 (51)	4113 (55)
Fifth quintile	5119 (5.2)	6385 (5.5)	2398 (47)	3255 (51)
**Marital status**				
Married/partnership	18655 (7.1)	24162 (7.6)	9738 (52)	13782 (57)
Unmarried/single	1858 (4.3)	2179 (4.7)	900 (48)	1187 (55)
Divorced	2389 (10)	3177 (10)	1389 (58)	1976 (62)
Widowed	5334 (13)	6942 (13)	3388 (64)	4610 (66)
**Smoking**				
Non-smoker	9152 (6.2)	11452 (6.6)	4774 (52)	6567 (43)
Former smoker	12007 (8.3)	16538 (9.0)	6605 (55)	9772 (59)
Current smoker	6058 (8.4)	6443 (9.3)	3391 (56)	3951 (61)
**Comorbidity over the last 12 months**				
Cancer	2108 (19)	NR	1378 (65)	NR
Headache/migraine	4940 (10)	NR	2779 (56)	NR
Neck/shoulder pain	6819 (17)	NR	4307 (63)	NR
Back pain	9163 (22)	NR	6272 (68)	NR
Arthrosis hip/knee	11895 (17)	NR	7467 (63)	NR
Rheumatoid arthritis/fibromyalgia	5274 (21)	NR	3509 (67)	NR

© 2019 Bedene A et al. *JAMA Network Open.*

eTable 1. Characteristics of Individuals in the 2012 and 2016 Dutch Health Monitor Surveys Who Received 1 or More Opioid Prescriptions (continued)

	2012 Rx, N02A n (%)	2016 Rx, N02A n (%)	2012 > 1 Rx, N02A n (%)	2016 > 1 Rx, N02A n (%)
**Feelings of depression**				
Always	467 (19)	627 (21)	322 (69)	454 (72)
Often	1442 (16)	1864 (17)	938 (65)	1302 (70)
Sometimes	4368 (12)	6186 (13)	2673 (61)	4027 (65)
Rarely	7591 (8.5)	10221 (8.8)	4287 (57)	6101 (60)
Never	13666 (6.0)	16617 (6.4)	6723 (49)	9064 (55)
**Feeling of loneliness, De Jong Gierveld scale**				
Not lonely (0-2 points)	13581 (6.3)	16285 (6.8)	6813 (50)	9051 (56)
Somewhat lonely (3-8 points)	9636 (8.4)	13026 (9.0)	5464 (57)	7917 (61)
Lonely (9-10 points)	2175 (12)	2908 (12)	1337 (62)	1886 (65)
Very lonely (11 points)	1326 (14)	1754 (14)	833 (62)	1170 (67)
**Able to make ends meet**				
No difficulties	9615 (6.0)	14036 (6.5)	4931 (51)	7845 (56)
Just able	10718 (7.7)	12973 (8.8)	5864 (57)	7687 (59)
Some difficulties	5141 (10)	5274 (12)	2942 (57)	3375 (64)
Great difficulties	2129 (14)	1955 (16)	1311 (62)	1323 (68)
**Miscellaneous**				
Heavy drinker[c]	1791 (5.9)	2304 (6.6)	869 (49)	1238 (54)
Lives alone	7533 (11)	10604 (11)	4658 (62)	6870 (65)
Unemployed	636 (7.6)	625 (7.3)	321 (54)	336 (54)
**Physical health**				
Very good/ good	11265 (4.1)	14318 (4.4)	4521 (40)	6613 (46)
Fair	12776 (14)	15866 (15)	7628 (60)	10068 (64)
Poor/ very poor	4880 (31)	6698 (32)	3630 (74)	5141 (77)
**Body mass index, kg/m2**				
< 18.5	506 (10)	588 (10)	323 (64)	433 (74)
18.5-20	768 (5.7)	963 (6.3)	419 (55)	599 (62)
20-25	9344 (5.9)	11105 (6.2)	4879 (52)	6345 (57)
25-30	11198 (7.8)	13788 (8.2)	5986 (54)	7868 (57)
> 30	6120 (12)	8662 (13)	3580 (59)	5417 (63)

N02A denotes ATC classification code for an opioid, Rx denotes prescription, NR denotes not reported
a MAVO, LBO (Dutch educational system)
b HAVO, VWO, MBO (Dutch educational system)
c 4(women)/6(men) glasses of alcohol per day at least once a week

© 2019 Bedene A et al. *JAMA Network Open.*

eTable 2. Opioid Prescription in the Netherlands Stratified by Age Group

	% of the population (% opioid use)			
	**2013**	**2014**	**2015**	**2016**
**Age group, years**				
15-25	12 (1.4)	12 (1.5)	12 (1.7)	12 (1.7)
25-45	26 (3.4)	26 (3.5)	25 (3.9)	25 (4.0)
45-65	28 (6.4)	28 (6.7)	28 (7.1)	28 (7.5)
> 65	17 (11.8)	17 (12.5)	18 (12.7)	18 (13.4)

Table shows individuals in the Netherlands, who reimbursed opioid prescription in the year of concern (2013-2016), stratified by age groups.

© 2019 Bedene A et al. *JAMA Network Open.*

eTable 3. Number of Individuals in the 2012 and 2016 Dutch Health Monitor Surveys Who Reimbursed 1, 2 to 4, or 5 or More Opioid Prescriptions

Opioid Prescriptions, n	2012 Rx N02A, n (%)	2016 Rx N02A, n (%)	2016 vs 2012 Rx N02A, OR (95% CI)[a]
1	13413 (45)	15291 (41)	1 (reference)
2-4	8928 (30)	12147 (32)	1.18 (1.14-1.23)
≥ 5	7212 (24)	10020 (27)	1.19 (1.15-1.25)

OR denotes odds ratio, CI denotes confidence interval
Total number of individuals, who reimbursed opioid prescriptions for the year 2012, n=29553 and for the year 2016, n=37458.
N02A denotes ATC classification code for an opioid, Rx denotes prescription
a Adjusted for age, sex, level of education, standardized household income and marital status.

© 2019 Bedene A et al. *JAMA Network Open.*

eTable 4. Characteristics of Individuals in the 2012 Dutch Health Monitor Survey Who Reimbursed 0, 1, 2 to 4, or 5 or More Opioid Prescriptions

	n (%)	0 Rx N02A, n (%)	1 Rx N02A, n (%)	2-4 Rx N02A, n (%)	≥ 5 Rx N02A, n (%)
Total	387195	357642 (92)	13413 (3.5)	8928 (2.3)	7212 (1.9)
**Sex**					
Men	175914	164907 (94)	5342 (3.0)	3297 (1.9)	2368 (1.3)
Women	211281	192735 (91)	8071 (3.8)	5631 (2.7)	4844 (2.3)
**Age group, years**					
19-35	53519	52027 (97)	977 (1.8)	361 (0.7)	154 (0.3)
35-45	43166	41209 (96)	1151 (2.7)	522 (1.2)	284 (0.7)
45-55	56275	52819 (94)	1788 (3.2)	983 (1.7)	685 (1.2)
55-65	60617	56137 (93)	2139 (3.5)	1372 (2.3)	969 (1.6)
> 65	162807	145322 (89)	7049 (4.3)	5476 (3.4)	4960 (3.0)
**Highest level of education**					
Primary school	37138	32058 (86)	1871 (5.0)	1608 (4.3)	1601 (4.3)
High school, underclassman[a]	131079	118692 (91)	5431 (4.1)	3820 (2.9)	3136 (2.4)
High School, upperclassman[b]	105863	99431 (94)	3236 (3.1)	1851 (1.7)	1345 (1.3)
College or more	99918	95722 (96)	2274 (2.3)	1208 (1.2)	714 (0.7)
**Immigration status**					
Native (Dutch)	335103	309922 (93)	11364 (3.4)	7579 (2.3)	6238 (1.9)
First generation	28163	25706 (91)	1178 (4.2)	790 (2.8)	489 (1.7)
Second generation	23927	22013 (92)	871 (3.6)	559 (2.3)	484 (2.0)
**Standardized household income**					
First quintile	39072	35413 (91)	1528 (3.9)	1117 (2.9)	1014 (2.6)
Second quintile	74437	66310 (89)	3119 (4.2)	2538 (3.4)	2470 (3.3)
Third quintile	82154	75501 (92)	3087 (3.8)	1983 (2.4)	1583 (1.9)
Fourth quintile	91375	85459 (94)	2913 (3.2)	1757 (1.9)	1246 (1.4)
Fifth quintile	97805	92686 (95)	2721 (2.8)	1510 (1.5)	888 (0.9)
**Marital status**					
Married/partnership	262953	244298 (93)	8917 (3.4)	5623 (2.1)	4115 (1.6)
Unmarried/single	42944	41086 (96)	958 (2.2)	498 (1.2)	402 (0.9)
Divorced	23424	21035 (90)	1000 (4.3)	765 (3.3)	624 (2.7)
Widowed	41069	35735 (87)	1946 (4.7)	1648 (4.0)	1740 (4.2)
**Smoking**					
Non-smoker	146773	137621 (94)	4378 (3.0)	2701 (1.8)	2073 (1.4)
Former smoker	144863	132856 (92)	5402 (3.7)	3716 (2.6)	2889 (2.0)
Current smoker	71818	65760 (92)	2667 (3.7)	1816 (2.5)	1575 (2.2)
**Comorbidity over the last 12 months**					
Cancer	11026	8918 (81)	730 (6.6)	621 (5.6)	757 (6.9)
Headache/ migraine	47634	42694 (90)	2161 (4.5)	1469 (3.1)	1310 (2.8)
Neck/ shoulder pain	39242	32423 (83)	2512 (6.4)	2153 (5.5)	2154 (5.5)
Back pain	42699	33536 (79)	2891 (6.8)	2937 (6.9)	3335 (7.8)
Arthrosis hip/ knee	72142	60247 (84)	4428 (6.1)	3731 (5.2)	3736 (5.2)
Rheumatoid arthritis/ fibromyalgia	24761	19487 (79)	1765 (7.1)	1619 (6.5)	1890 (7.6)

© 2019 Bedene A et al. *JAMA Network Open.*

eTable 4. Characteristics of Individuals in the 2012 Dutch Health Monitor Survey Who Reimbursed 0, 1, 2 to 4, or 5 or More Opioid Prescriptions (continued)

	n (%)	0 Rx N02A, n (%)	1 Rx N02A, n (%)	2-4 Rx N02A, n (%)	≥ 5 Rx N02A, n (%)
**Feelings of depression**					
Always	2404	1937 (81)	145 (6.0)	148 (6.2)	174 (7.2)
Often	8979	7537 (84)	504 (5.6)	434 (4.8)	504 (5.6)
Sometimes	36730	32362 (88)	1695 (4.6)	1331 (3.6)	1342 (3.7)
Rarely	89587	81996 (92)	3304 (3.7)	2298 (2.6)	1989 (2.2)
Never	227577	213911 (94)	6943 (3.1)	4098 (1.8)	2625 (1.2)
**Feeling of loneliness, De Jong Gierveld scale**					
Not lonely (0-2 points)	216407	202826 (94)	6768 (3.1)	3982 (1.8)	2831 (1.3)
Somewhat lonely (3-8 points)	114222	104586 (92)	4172 (3.7)	3012 (2.6)	2452 (2.1)
Lonely (9-10 points)	17980	15805 (88)	838 (4.7)	685 (3.8)	652 (3.6)
Very lonely (11 points)	9604	8278 (86)	493 (5.1)	375 (3.9)	458 (4.8)
**Able to make ends meet**					
No difficulties	160284	150669 (94)	4684 (2.9)	2835 (1.8)	2096 (1.3)
Just able	138441	127723 (92)	4854 (3.5)	3290 (2.4)	2574 (1.9)
Some difficulties	49777	44636 (90)	2199 (4.4)	1543 (3.1)	1399 (2.8)
Great difficulties	15094	12965 (86)	818 (5.4)	657 (4.4)	654 (4.3)
**Miscellaneous**					
Heavy drinker[c]	30585	28794 (94)	922 (3.0)	566 (1.9)	303 (1.0)
Lives alone	70210	62677 (89)	2875 (4.1)	2340 (3.3)	2318 (3.3)
Unemployed	8369	7733 (92)	315 (3.8)	209 (2.5)	112 (1.3)
**Physical health**					
Very good/ good	276830	265565 (96)	6744 (2.4)	3215 (1.2)	1306 (0.5)
Fair	89435	76659 (86)	5148 (5.8)	4082 (4.6)	3546 (4.0)
Poor/ very poor	15943	11063 (69)	1250 (7.8)	1449 (9.1)	2181 (13.7)
**Body mass index, kg/m2**					
< 18.5	5061	4555 (90)	183 (3.6)	142 (2.8)	181 (3.6)
18.5-20	13845	12717 (94)	349 (2.5)	195 (1.4)	224 (1.6)
20-25	159525	150181 (94)	4465 (2.8)	2689 (1.7)	2190 (1.4)
25-30	143098	131900 (92)	5212 (3.6)	3461 (2.4)	2525 (1.8)
> 30	50639	44519 (88)	2540 (5.0)	1976 (3.9)	1604 (3.2)

N02A denotes ATC classification code for an opioid, Rx denotes prescription
a MAVO, LBO (Dutch educational system)
b HAVO, VWO, MBO (Dutch educational system)
c 4(women)/6(men) glasses of alcohol per day at least once a week

© 2019 Bedene A et al. *JAMA Network Open.*
